# Prevalence of Metabolic Syndrome in Women After Maternal Complications of Pregnancy: An Observational Cohort Analysis

**DOI:** 10.3389/fcvm.2022.853851

**Published:** 2022-03-14

**Authors:** Emily Aldridge, Maleesa Pathirana, Melanie Wittwer, Susan Sierp, Shalem Y. Leemaqz, Claire T. Roberts, Gustaaf A. Dekker, Margaret A. Arstall

**Affiliations:** ^1^Adelaide Medical School, University of Adelaide, Adelaide, SA, Australia; ^2^Robinson Research Institute, University of Adelaide, Adelaide, SA, Australia; ^3^Department of Cardiology, Northern Adelaide Local Health Network, Elizabeth Vale, SA, Australia; ^4^Flinders Health and Medical Research Institute, Flinders University, Bedford Park, SA, Australia; ^5^Department of Obstetrics & Gynecology, Northern Adelaide Local Health Network, Elizabeth Vale, SA, Australia

**Keywords:** metabolic syndrome, pregnancy complications, cardiovascular disease, cardiovascular disease prevention, women

## Abstract

**Introduction:**

Certain complications of pregnancy, including hypertensive disorders of pregnancy, gestational diabetes mellitus, intrauterine growth restriction, spontaneous preterm birth, and placental abruption, are established independent risk factors for premature cardiovascular disease in women. Metabolic syndrome, which is associated with an increased risk of cardiovascular disease, may be a suitable alternative to traditional cardiovascular risk calculators that underestimate risk in young women. This study aimed to investigate the prevalence of metabolic syndrome in women who experienced a complicated pregnancy 6 months earlier.

**Methods:**

This observational study investigated the prevalence of metabolic syndrome as defined by the International Diabetes Federation in all eligible participants (*n* = 247) attending a postpartum lifestyle intervention clinic from August 2018 to June 2021 at the Lyell McEwin Hospital in Adelaide, South Australia.

**Results:**

A total of 89 (36%) participants met the criteria for metabolic syndrome at a mean follow up time of 7 months postpartum. Almost 90% of the cohort were abdominally obese, and over two thirds of the total cohort met at least two of the criteria for metabolic syndrome.

**Conclusions:**

Women with a prior history of one of the common major pregnancy complications are at high risk of future cardiovascular and metabolic disease, with many showing either metabolic syndrome or multiple risk factors at only 7 months postpartum. The results indicate that follow-up within 1 year postpartum is an appropriate time to commence preventative strategies, as many women are already showing early signs of disease.

## Introduction

Cardiovascular disease (CVD) continues to be the leading cause of death for women worldwide, and rates of CVD and wstroke are increasing in women aged <45 years (1, 2). Certain complications of pregnancy, including hypertensive disorders of pregnancy, gestational diabetes mellitus, intrauterine growth restriction, spontaneous preterm birth, and placental abruption, are established independent risk factors for premature cardiovascular disease in women (3–9). Despite this, awareness of the increased risk of CVD for women who have experienced at least one of these complications of pregnancy remains low (10, 11).

In 2011, the American Heart Association and the European Society of Cardiology updated their guidelines to include recommendations for monitoring women with a previous history of any of these complications (12, 13), an important step in recognizing the risk posed to this specific population of high-risk women. The Maternal Health Clinic in Ontario, Canada, was introduced in 2011 and is the first postpartum intervention for women with a recent history of complicated pregnancies, inviting eligible women to attend lifestyle education at 6 months postpartum (14). This model has been adopted across a number of sites worldwide in the last decade, and in recent years, a number of research-based postpartum interventions have also been developed. However, these research-based interventions are often opt-in research studies rather than routine outpatient care, tend to focus on specific complications of pregnancy, such as preeclampsia (15–18), and the effectiveness of these interventions at reducing cardiovascular and metabolic disease risk has not been rigorously assessed in a real-world clinic setting. The first nurse-led postpartum clinic in Australia was introduced in 2018 at the Lyell McEwin Hospital and aims to provide structured lifestyle and risk education (19). To our knowledge, this is the only published and freely available nurse-led model of care in Australia of standardized, routine, ongoing outpatient care for all eligible women who have experienced a serious complication of pregnancy. In most areas of Australia and the world, there remains little to no attention paid to women in this cohort, despite the recommendations for follow-up in national and international guidelines.

In the minority of women who do receive follow up after a complicated pregnancy, there remains the difficulty of effectively communicating risks to women. The currently available cardiovascular risk scores have been developed using acute coronary syndrome thresholds found in predominantly male populations, reducing their reliability for use in women (20). The vast majority also fail to consider early changes in cardiovascular measures as well as the young age of the study population, which conceals the high-risk, long-term impact of a complication of pregnancy (21). The Absolute Cardiovascular Disease Risk Calculator, for example, is commonly used by general practitioners in Australia for calculating cardiovascular risk in adults, and is recommended by the Australian Heart Foundation. However, there is minimal evidence supporting its use in people aged less than 30 years old, and limited strong evidence for people aged 30–45 years (22). Current cardiovascular risk calculators are therefore not appropriate tools for detecting subclinical disease in and communicating risk to young women. A lack of a clear and effective way to explain risk to women may reduce the impact of preventative strategies.

Metabolic syndrome is defined as a ‘cluster of the most dangerous heart attack risk factors: diabetes and prediabetes, abdominal obesity, high cholesterol and high blood pressure' (23, 24). Metabolic syndrome is associated with a 4-fold higher risk of developing cardiovascular disease in women and 2-fold higher risk in men (25). Considering the lack of availability of appropriate cardiovascular risk scores for younger women, metabolic syndrome may be an appropriate alternative for assessing and communicating the high long-term risk to women who are at low short-term risk of CVD. However, the prevalence of metabolic syndrome in young women is not well-documented, especially in socioeconomically disadvantaged cohorts where it may be higher (26). Therefore, investigating the prevalence of metabolic syndrome is the first step in understanding the risk of future cardiovascular disease in these populations.

The primary objective of this research is to investigate the prevalence of metabolic syndrome in a cohort of women who experienced a severe maternal complication of pregnancy 6 months earlier. The secondary objectives were to explore the presence of socioeconomic, metabolic, and cardiovascular risk factors in this cohort.

## Methods

### Study Design

This was an observational study of women attending the postpartum lifestyle intervention clinic from 7th August 2018 to 30th June 2021 at the Lyell McEwin Hospital, located within the Northern Adelaide Local Health Network (NALHN), South Australia (19). The Central Adelaide Local Health Network Human Research Ethics Committee approved the study [HREC/16/TQEH/258].

### Study Participants

To be eligible for referral to the postpartum intervention clinic, patients must have experienced at least one of the following complications in their index pregnancy:

- Hypertensive disorders of pregnancy (including gestational hypertension, preeclampsia, eclampsia and HELLP syndrome), requiring medical wtherapy or resulting in birth <37 weeks' gestation. Hypertensive disorders were diagnosed according to criteria defined by the International Society for the Study of Hypertension in Pregnancy (27).
- Gestational diabetes mellitus, diagnosed according to HAPO criteria (28, 29) requiring metformin or insulin therapy.
- Spontaneous preterm birth <34 weeks' gestation.
- Intrauterine growth restriction indicated by serial ultrasound measurements, estimated fetal weight, and umbilical artery doppler, or delivery of a small for gestational age infant at <5th customized birth centile, as per South Australian Perinatal Practice Guidelines (30, 31).
- Placental abruption.

### Study Setting

The Lyell McEwin Hospital is a public tertiary acute-care facility providing obstetric care, adult cardiac and intensive care services, and neonatal care for infants for ≥32 weeks' gestation. Located within the NALHN catchment area, the Lyell McEwin Hospital services a population of approximately 400,000 people in the northern and north-eastern suburbs of Adelaide, South Australia. The NALHN area is characterized by a population with low socioeconomic status with high rates of CVD morbidity and mortality, and is among Australia's most disadvantaged suburban communities (32).

### Study Procedures

The postpartum intervention is a hospital-based outpatient clinical service with an associated quality assurance registry, the methods of both have been previously described (19). Briefly, eligible patients are offered appointments at approximately 6 and 18 months postpartum to undergo a thorough health assessment and receive individualized health counseling from an expert nurse practitioner. Variables are collected from a combination of patient self-report and abstraction from the hospital medical record. Information collated and included in the registry includes patient demographics, medical history, family history, current medications, alcohol, drug and smoking practices, obstetric history, cardiovascular and metabolic screening pathology results, peripheral and central blood pressure, augmentation index, pulse rate, height, weight, and waist circumference. Blood pressure, augmentation index, and pulse rate were measured using an oscillometric pulse wave analysis device, the USCOM BP+ [USCOM, Sydney, Australia]. This device has been previously validated against aneroid blood pressure measurements (33).

### Outcomes

The primary outcome of interest for this study was prevalence of metabolic syndrome at approximately 6-months postpartum.

Metabolic syndrome was defined as the presence of any three of the following five risk factors (24):

- Elevated waist circumference with ethnicity specific values defined by the International Diabetes Federation (23), which for women is ≥80 cm for all ethnicities.
- Elevated triglycerides of ≥1.7 mmol/L, or drug treatment for this lipid abnormality.
- Reduced HDL cholesterol of <1.3 mmol/L, or drug treatment for this lipid abnormality.
- Elevated systolic blood pressure of ≥130 mmHg and/or diastolic blood pressure of ≥85 mmHg, or antihypertensive drug treatment.
- Elevated fasting glucose of ≥5.6 mmol/L, or drug treatment of elevated glucose.

Secondary outcomes were individual cardiovascular and metabolic risk factors and included current smoking, waist circumference, BMI, peripheral and central systolic blood pressure, peripheral and central diastolic blood pressure, pulse rate, augmentation index, triglycerides, HDL cholesterol, fasting plasma glucose, and fasting insulin. Demographic outcomes recorded included ethnicity, country of birth, preferred language, level of education, employment status, marital status, and combined household income. Area-level socioeconomic status was recorded using the Socio-Economic Indexes for Areas (SEIFA), specifically the Index of Relative Socioeconomic Advantage and Disadvantage (IRSAD).

### Analysis

Continuous variables are presented as mean and standard deviation for normally distributed variables, and median and interquartile range for non-normally distributed variables. Categorical data are presented as count and percentage. Participants with metabolic syndrome at 6 months postpartum were compared to the reference group, which was comprised of participants without metabolic syndrome. As this study presented purely descriptive data and no inferences could be made, no statistical tests were performed. All descriptive analyses were conducted using IBM SPSS Statistics for Windows, version 27.0 (Armonk, NY: IBM Corp).

Participants were only included in this study if their metabolic syndrome status could be confirmed. Therefore, those with abdominal obesity who did not complete biochemistry were excluded, as metabolic syndrome status could not be confirmed. Participants who were pregnant again at the time of their appointment were also excluded due to the inability to accurately determine abdominal obesity and the influence of the pregnancy on blood pressure, maternal lipids, and glucose.

### COVID-19 Considerations

During the study period, the postpartum intervention clinic was required to close for two separate periods, firstly between 1st April 2020 and 20th May 2020, and then again between 20th and 27th of July 2021, due to COVID-19 lockdowns. During these periods, appointments were unable to take place and patients with scheduled appointments were planned to be rescheduled upon the clinic reopening. Online and telehealth consults were not offered due to the impossibility of obtaining accurate anthropometric measurements (including blood pressure, height, weight, and waist circumference) and performing physical assessments.

## Results

A total of 517 eligible women were offered appointments at the postpartum intervention clinic during the study timeframe. A total of 261 participants (50.4%) attended appointments and were included in the registry. Ten participants were excluded from the present analysis as they had not completed their biochemistry testing and metabolic syndrome status was unable to be determined. Another four participants were excluded due to being pregnant at their first postpartum appointment. This resulted in a final cohort of 247 for analysis in the present study.

Descriptive statistics for metabolic measures and socioeconomic factors at time of appointment are presented in Table 1. The mean time to follow up was 7 months postpartum, with a range of 4–21 months. Delayed appointments were caused by numerous factors, including: clinic closure due to COVID-19, multiple rescheduled appointments due to maternal request/illness, missed or late referrals to the service, and administrative issues.

**Table 1:** Maternal characteristics according to maternal metabolic syndrome status at first postpartum appointment.

**Variable**	**Value, mean** **±SD or n (%)**					
	**Total cohort**	**Total, *n***	**MetS *n* = 89 (36%)**	**Total, *n***	**No MetS *n* = 158 (64%)**	**Total, *n***
Time to follow-up, months	7.13 ± 2.2	247	7.4 ± 2.1	89	7.0 ± 2.3	158
Age, years	32.8 ± 5.2	247	33.3 ± 5.2	89	32.6 ± 5.2	158
Gravidity at time of referral, n	2.8 ± 1.7	247	3 ± 2.0	89	2.7 ± 1.6	158
Pregnancy booking BMI, kg/m^2^	30.6 ± 8.0	242	33.8 ± 7.7	88	28.7 ± 7.7	154
**Cardiovascular and metabolic risk factors**						
BMI, kg/m^2^	31.62 ± 7.9	247	35.8 ± 7.9	89	29.3 ± 6.9	158
Waist circumference, cm	98.30 ± 17.7	246	107.8 ± 16.6	89	92.9 ± 15.9	157
Peripheral SBP, mmHg	123 ± 13	245	127 ± 13	88	120 ± 12	157
Peripheral DBP, mmHg	74 ± 10	245	78 ± 10	88	72 ± 10	157
Central SBP, mmHg	116.0 ± 13.5	238	120.6 ± 13.0	86	113.4 ± 13.1	152
Central DBP, mmHg	76.6 ± 10.5	238	80.4 ± 10.0	86	74.36 ± 10.1	152
Pulse rate, bpm	74.6 ± 11.0	245	77.1 ± 12.2	88	73.2 ± 10.0	157
Augmentation index, %	76.4 ± 28.9	238	77.5 ± 28.5	86	75.8 ± 28.5	152
Triglycerides, mmol/L	1.33 ± 0.8	242	1.9 ± 0.8	88	1.0 ± 0.6	154
HDL cholesterol, mmol/L	1.37 ± 1.0	241	1.1 ± 0.2	88	1.5 ± 1.2	153
Glucose, mmol/L	5.24 ± 1.34	240	5.7 ± 1.5	86	5.0 ± 1.2	154
Insulin, μ/L	15.60 ± 13.7	237	23.4 ± 1.7	86	11.2 ± 10.1	151
Current smoking	23 (9.3)	247	10 (11.2)	89	13 (8.2)	158
T2DM	11 (4.5)	247	8 (9.0)	89	3 (1.9)	158
T1DM	4 (1.6)	247	0 (0)	89	4 (2.5)	158
Hypertension	16 (6.5)	247	13 (14.6)	89	3 (1.9)	158
**Demographics**						
Born in Australia	133 (53.8)	247	48 (53.9)	89	85 (53.8)	158
Interpreter required	43 (17.4)	247	16 (18.0)	89	27 (17.1)	158
Ethnicity		247		89		158
*Caucasian* *Chinese & other Asian* *Middle eastern* *Indian subcontinent* *African* *Hispanic* *Aboriginal*	135 (54.7) 50 (20.2) 22 (8.9) 20 (8.1) 14 (5.7) 2 (0.8) 4 (1.6)		49 (55.1) 20 (22.5) 9 (10.1) 7 (7.9) 2 (2.2) 1 (1.1) 1 (1.1)		86 (54.4) 30 (19.0) 13 (8.2) 13 (8.2) 12 (7.6) 1 (0.6) 3 (1.9)	
Marital status		247		89		158
*Married* *De facto* *Single* *Relationship, not living together* *Separated*	174 (70.4) 49 (19.8) 19 (7.7) 4 (1.6) 1 (0.4)		61 (68.5) 17 (19.1) 10 (11.2) 1 (1.1) 0 (0)		113 (71.5) 32 (20.3) 9 (5.7) 3 (1.9) 1 (0.6)	
Level of education		247		89		158
*Unknown* * < = Year 10* *Year 11* *Year 12* *TAFE certificate/diploma* *Bachelor* *Higher degree*	27 (10.9) 15 (6.1) 12 (4.9) 33 (13.4) 96 (38.9) 48 (19.4) 16 (6.5)		13 (14.6) 6 (6.7) 2 (2.2) 14 (15.7) 36 (40.4) 14 (15.7) 4 (4.5)		14 (8.9) 9 (5.7) 10 (6.3) 19 (12.0) 60 (38.0) 34 (21.5) 12 (7.6)	
Currently employed	130 (53.8)	247	37 (41.6)	89	93 (58.9)	158
Type of employment		247		89		158
*Maternity leave* *Full-time* *Part-time* *Casual* *Student* *None*	73 (29.6) 8 (3.2) 25 (10.1) 22 (8.9) 2 (0.8) 117 (47.4)		21 (23.6) 5 (5.6) 4 (4.5) 6 (6.7) 1 (1.1) 52 (58.4)		52 (32.9) 3 (1.9) 21 (13.3) 16 (10.1) 1 (0.6) 65 (41.1)	
Annual household income		247		89		158
*$205,000* *$105–205,000* *$70–100,000* *$40–70,000* *$20–40,000* * < = $20,000* *Unknown or declined to answer*	2 (0.8) 39 (15.8) 54 (21.9) 43 (17.4) 15 (6.1) 9 (3.6) 85 (34.4)		0 (0) 9 (10.1) 21 (23.6) 16 (18.0) 4 (4.5) 4 (4.5) 35 (39.3)		2 (1.3) 30 (19.0) 33 (20.9) 27 (17.1) 11 (7.0) 5 (3.2) 50 (31.6)	
SEIFA - IRSAD	920.5 ± 65.4	247	916.2 ± 69.9	89	922.9 ± 62.7	158

*SD, standard deviation; MetS, metabolic syndrome; BMI, body mass index; SBP, systolic blood pressure; DBP, diastolic blood pressure; HDL, high-density lipoprotein; T2DM, type 2 diabetes mellitus; T1DM, type 1 diabetes mellitus; TAFE, technical and further education; SEIFA, socioeconomic index for area; IRSD, index of relative socioeconomic disadvantage; IRSAD, index of relative socioeconomic advantage and disadvantage; IEO, index of education and occupation; IER, index of economic resources*.

Overall, 89 (36%) of the participants met the criteria for metabolic syndrome. The mean age of women seen in the postpartum intervention clinic was 32.8 years, with a BMI of 31.62 kg/m^2^. Sixteen and eleven women had been diagnosed with hypertension and type 2 diabetes prior to their first postpartum appointment, respectively. Half of the cohort was Caucasian and born in Australia, and most were married or in de facto relationships.

When comparing those women with and without metabolic syndrome, there were differences in all metabolic measures, as expected. There was also a higher number of participants with previously diagnosed type 2 diabetes and hypertension in the metabolic syndrome group. There were no differences in socioeconomic measures between groups, excepting employment status. Employment rates were higher in participants that did not have metabolic syndrome (58.9%) vs. those with metabolic syndrome (41.6%).

Table 2 presents metabolic and cardiovascular risk factors for the total cohort and comparing between metabolic syndrome status groups. Overall, 89.5% of the cohort had abdominal obesity at the time of their appointment, and over 90% of the cohort fulfilled at least one criterion for metabolic syndrome (including, but not necessarily meeting the abdominal obesity requirement).

**Table 2:** Metabolic syndrome and cardiovascular risk factors present in total cohort.

**Variable**	**Value, n (%)**					
	**Whole cohort**	**Total, *n***	**MetS**	**Total, *n***	**No MetS**	**Total, *n***
Abdominal obesity: add BMI + WC	221 (89.5)	247	89 (100)	89	132 (83.5)	158
Reduced HDL cholesterol: <1.29 mmol/L or treatment for this lipid abnormality	113 (46.8)	241	76 (86.4)	88	37 (24.2)	153
Raised triglycerides: ≥1.7 mmol/L or treatment for this lipid abnormality	62 (25.6)	242	54 (61.4)	88	8 (5.2)	154
Raised fasting plasma glucose: ≥5.6 mmol/L or treatment for T2DM	48 (19.8)	242	39 (44.3)	88	9 (5.8)	154
**Additional risk factors**						
Raised fasting plasma glucose: ≥5.6 mmol/L, treatment for T2DM or raised fasting insulin: ≥12 μ/L	132 (55.5)	238	77 (88.5)	87	55 (36.4)	151
Treated hypertension, SBP ≥135mmHg or DBP ≥85mmHg	75 (30.6)	245	48 (54.5)	88	27 (17.2)	157
Current smoking	23 (9.3)	247	10 (11.2)	89	13 (8.2)	158
Number of MetS risk factors present^*^		243		89		154
0 1 2 3 4 5	16 (6.6) 63 (25.9) 76 (31.3) 54 (22.2) 27 (11.1) 7 (2.9)		0 (0) 0 (0) 1 (1.1) 54 (60.7) 27 (30.3) 7 (7.9)		16 (10.4) 63 (40.9) 75 (48.7) 0 (0) 0 (0) 0 (0)	

* *Includes any one criterion for metabolic syndrome, whether or not abdominal obesity is present*.

*MetS, metabolic syndrome; HDL, high-density lipoprotein; T2DM, type 2 diabetes mellitus; SBP, systolic blood pressure; DBP, diastolic blood pressure*.

The breakdown of referring pregnancy complications is presented in Table 3. There were no differences between the metabolic syndrome group and the group without metabolic syndrome, although there was a slightly higher percentage of gestational diabetes in the metabolic syndrome group. Gestational diabetes was the most frequent reason for referral to the clinic (71.7%), followed by hypertensive disorders of pregnancy (32.4%).

**Table 3:** Index pregnancy complications of cohort^*^ (*n* = 247).

**Variable**	**Value**, ***n*** **(%)**		
	**Total cohort** ***N* = 247**	**MetS** ***N* = 89**	**No MetS** ***N* = 158**
Hypertensive disorder of pregnancy	80 (32.4)	33 (37.1)	47 (29.7)
Gestational diabetes	177 (71.7)	70 (78.7)	107 (67.7)
Placental abruption	4 (1.6)	1 (1.1)	3 (1.9)
Intrauterine growth restriction	17 (6.9)	4 (4.5)	13 (8.2)
Spontaneous preterm birth	5 (2.0)	2 (2.2)	3 (1.9)

* *Participants may have experienced more than one complication; the pregnancy complication categories are not mutually exclusive. These complications include only those listed on the referral, which is made at time of discharge from delivery of infant. Only complications experienced in the index pregnancy were considered during the referral process*.

*MetS, metabolic syndrome*.

## Discussion

In this prospective cohort of 247 participants with a major prior pregnancy complication, over one-third (36%) of the cohort met the criteria for metabolic syndrome 6months after pregnancy. This indicates that these young women are at detectably high risk of future cardiovascular disease. Worryingly, two-thirds (67.5%) of the whole cohort met at least two of the metabolic syndrome criteria, and 89.5% had abdominal obesity with a BMI >30 kg/m^2^ and/or waist circumference ≥80 cm, irrespective of metabolic syndrome. In the group without metabolic syndrome, 89.6% met at least one of the metabolic syndrome criteria. This suggests that, even in those without metabolic syndrome, the vast majority of women in this cohort are at least obese and therefore on a trajectory to additional cardiovascular and metabolic disease risk. Although metabolic syndrome status prior to pregnancy was not able to be determined, pregnancy booking BMI, taken at the time of the antenatal triage appointment in the first trimester, was significantly higher in the group of participants who had metabolic syndrome postpartum (33.8 kg/m^2^) vs. those without (28.7 kg/m^2^), suggesting that metabolic syndrome was likely already present in a number of the cohort prior to conception.

Previous studies have found that women with a history of pregnancy complications show increased risk and subclinical signs of chronic disease early after pregnancy. The prevalence of metabolic syndrome in this study was more than twice that found in a Canadian study by Cusimano and colleagues, which reported that 17.4% of women had metabolic syndrome at 6 months postpartum following a complicated pregnancy (34). Their cohort included women of any gravidity with the same complications of pregnancy who were included in our study, and therefore their results are directly comparable to those presented in this analysis. The disparity in prevalence of metabolic syndrome between cohorts demonstrate how risk and health status may vary greatly between populations, emphasizing the need for analyzing and understanding the risk profile of local cohorts when introducing preventative strategies.

There are some previous studies exploring metabolic syndrome both in pregnancy and after pregnancy complications. A study of the Screening for Pregnancy Endpoints (SCOPE) cohort found that 29.4% of nulliparous women recruited from the Adelaide, South Australia, site had metabolic syndrome at 15 weeks' gestation (35). These participants were recruited in 2005–2008 from the same hospital as the cohort in the present study. Although the study included healthy nulliparous women without pre-existing hypertension and/or diabetes, most of whom went on to have an uncomplicated pregnancy, the lower percentage of metabolic syndrome reported in a very similar population could suggest that rates may have increased over the last 15 years. The higher prevalence of metabolic syndrome in our cohort also suggests that we are referring the right individuals for further follow up and education.

Another study found 34% of women with a history of hypertensive disorders of pregnancy had metabolic syndrome 2.5 years after the index pregnancy, compared to only 5% of those who had had a normotensive pregnancy (36). This is a similar percentage to our cohort (36%) but our women were followed up much earlier in the postpartum period. It is possible that, especially without intervention, we would see an increase in the rate of metabolic syndrome in our cohort over time. However, Smith and colleagues followed women with a history of preeclampsia and found that the prevalence of metabolic syndrome did not change from 1 to 3 years postpartum (37). Further research should explore the progression of metabolic syndrome over time, especially in women with a history of the complications not characterized by hypertension. Although the current study was not able to address progression of metabolic syndrome without intervention, the results indicate that due to a high prevalence of metabolic syndrome at 6 months postpartum, commencing follow-up and education strategies within 1 year postpartum is appropriate.

In the existing literature, less attention has been paid to the rarer complications of pregnancy, including intrauterine growth restriction, spontaneous preterm birth, and placental abruption. Underweight women are at higher risk of having a growth restricted infant (38), and would therefore not meet the essential abdominal obesity criterion of metabolic syndrome. However, one study exploring women with previous delivery of a small for gestational age infant reported 7.6% had metabolic syndrome (as defined by the World Health Organization) after pregnancy (39). Another small study found that women with metabolic syndrome early in pregnancy were almost three times more likely to deliver preterm compared to those without metabolic syndrome (40). Our study was unable to show any differences in metabolic syndrome prevalence between complications of pregnancy due to small numbers, but further research in these groups should be conducted.

This study also explored differences in individual- and area-level socioeconomic factors between those with metabolic syndrome and those without, although there were no noticeable differences in any of these except for employment status. Although women with metabolic syndrome were less likely to be employed (41.6) compared to those without metabolic syndrome (58.9), the data may not capture an accurate reflection of employment history, as some women may have been previously employed but recently ceased working due to caring responsibilities for their new infant. We did not collect data on future employment plans or recent employment history. There were also missing data in education (10.9%) and income variables, with 34.4% of the cohort either declining to answer or reporting not knowing their household income. Previous literature has reported a relationship between low socioeconomic status (determined using one or a combination of level of education, income, and employment) and higher incidence of metabolic syndrome, especially for women (41–45). The lack of differences in the present analysis may be due to the small sample size or the heterogeneity of the participants. Further socioeconomic analyses of this cohort will be conducted as more women are included in the quality assurance registry.

The high prevalence of metabolic syndrome, and the high percentage of modifiable metabolic and cardiovascular disease risk factors, in our cohort provide justification for the need for early postpartum intervention with ongoing follow up for women who experience complicated pregnancies. In the literature, it is evident that many women remain unaware of the risk posed to them after having a complicated pregnancy. Follow up for cardiovascular risk screening after hypertensive disorders of pregnancy remains minimal (46), with most women reporting low personal awareness of cardiovascular disease risk (11). Even after being informed of their increased risk, most women attending the Maternal Health Clinic in Canada continued to underestimate their risk (10). Furthermore, women with gestational diabetes also underestimate their risk of developing type 2 diabetes (47), generally tend to not adopt healthy lifestyles postpartum (48), and report a number of barriers to making healthy lifestyle changes (49). It is therefore vital to provide structured follow up care for these patients to encourage positive lifestyle modifications as soon as possible.

The attendance rate to the intervention clinic was only just over 50% during the study timeframe, and the current analysis did not capture data from women who did not attend their appointment, so inclusion bias in the present study is highly likely. Although not explored in this analysis, previous research from the Maternal Health Clinic in Canada found that women choosing not to attend had more adverse metabolic and social risk profiles than those who did attend (50). This could indicate that the women in our population most in need of intervention are not engaging with preventative health services. Future research into how to improve engagement is required. Introduction of positive health messages during pregnancy and ideally prior to conception may be of additional benefit. A previous systematic review found that dietary and lifestyle interventions introduced during pregnancy have the potential for reducing the risk of developing preeclampsia (51).

Another factor that may have influenced the attendance rate is the COVID-19 pandemic. COVID-19 lockdowns resulted in the postpartum intervention clinic closing for two separate periods during the study. Although women were re-offered appointments upon reopening, we expect that attendance was at least in part influenced by general concerns about attending healthcare facilities during the pandemic, when the general population were strongly encouraged to avoid leaving their homes unless absolutely necessary. Since 2022, online consultations have been offered by the postpartum clinic due to ongoing pandemic restrictions on outpatient services, however online or telehealth consults introduce further difficulties as measurements and physical assessments cannot be performed. Remote consults are also impractical for non-English speaking patients who require an interpreter.

There are several limitations of the present study that warrant discussion. The postpartum clinic's eligibility criteria are currently limited to the most severe examples of complications of pregnancy and do not include women with unmedicated hypertensive disorders of pregnancy or gestational diabetes. If unmedicated participants were also included, the overall prevalence of metabolic syndrome may have been reduced. Furthermore, there are no data available on the prevalence of metabolic syndrome in women with healthy pregnancies or in the wider population of young women from which our cohort was recruited, and so the prevalence reported in this study may be reflective of the broader profile of our population. Only prospective data were recorded at the time of the postpartum appointment, so no historical socioeconomic information (for example, the socioeconomic status the women were raised in) were available. Finally, the data in this study were collected from a unique outpatient service in a highly disadvantaged community, and therefore the prevalence of metabolic syndrome may not reflect that of other settings.

A strength of this study included using complete metabolic syndrome diagnostic criteria instead of traditional cardiovascular risk calculators to provide a more accurate reflection of risk. We were also able to collect a comprehensive dataset for inclusion in the registry, which will continue to collect data for future in-depth analyses as this cohort grows.

## Conclusion

The results of this study highlight the need for ongoing follow-up services for women who have had a complication of pregnancy, as they are at high risk of future metabolic and cardiovascular disease. Pregnancy and the early postpartum period are opportune times to engage with high-risk women about their increased risk due to increased contact with the healthcare system. Future research should focus on improving engagement with postpartum interventions, exploring the effectiveness of preventative services in the reduction of metabolic syndrome, and development of other methods of calculating cardiovascular risk in this disadvantaged population.

## Data Availability Statement

The datasets generated for this study will not be made publicly available as the authors are not permitted to share datasets due to ethical requirements. Requests to access the datasets should be directed to Emily Aldridge, emily.aldridge@adelaide.edu.au.

## Ethics Statement

This study involving human participants was reviewed and approved by Central Adelaide Local Health Network Human Research Ethics Committee (CALHN HREC). The Ethics Committee waived the requirement of written informed consent for participation.

## Author Contributions

EA designed the study, collected the data, performed data analysis, and prepared the manuscript. MP and SS assisted with data collection. SYL provided statistical analysis support. CTR, GAD, and MAA supervised the study. All authors edited the draft manuscript, provided critical feedback, and approved the final manuscript.

## Funding

EA was supported by a fellowship awarded by The Hospital Research Foundation (Grant ID 2018/006-QA25232).

## Conflict of Interest

The authors declare that the research was conducted in the absence of any commercial or financial relationships that could be construed as a potential conflict of interest.

## Publisher's Note

All claims expressed in this article are solely those of the authors and do not necessarily represent those of their affiliated organizations, or those of the publisher, the editors and the reviewers. Any product that may be evaluated in this article, or claim that may be made by its manufacturer, is not guaranteed or endorsed by the publisher.
